# Therapeutic Deep Eutectic Systems towards the Treatment of Tuberculosis and Colorectal Cancer: Opportunities and Challenges

**DOI:** 10.3390/molecules26227022

**Published:** 2021-11-20

**Authors:** Filipe Oliveira, Filipa Santos, Ana Rita C. Duarte

**Affiliations:** Associated Laboratory for Green Chemistry (LAQV) of the Network of Chemistry and Technology (REQUIMTE), Chemistry Department, NOVA School of Science and Technology, 2829-516 Caparica, Portugal; fsn.oliveira@campus.fct.unl.pt (F.O.); mfca.santos@campus.fct.unl.pt (F.S.)

**Keywords:** therapeutic deep eutectic systems, active pharmaceutical ingredient, tuberculosis, colorectal cancer, drug delivery

## Abstract

What if a new technology based on therapeutic deep eutectic systems would disrupt the current treatment of major economic and socially burden diseases? The classical definition of eutectic systems is that they are the combination of two or more compounds that interact via hydrogen bonds, from which results a melting temperature depression in comparison with that of its individual components. Therapeutic deep eutectic systems are defined as eutectic systems in which at least one of the individual components is an active pharmaceutical ingredient, or a eutectic system in which the active pharmaceutical ingredient is dissolved. Current literature reports on tuberculosis have been mostly based on the most common anti-tuberculosis drugs prescribed. Using eutectic systems based on naturally occurring molecules known for their anti-microbial activity may also present a promising therapeutic strategy able to cope with the prevalence of *Mycobacterium tuberculosis* and prevent the appearance of multidrug resistance strains. With regards to colorectal cancer, literature has been unravelling combinations of terpenes with anti-inflammatory drugs that are selectively cytotoxic towards colorectal cancer cells and do not compromise the viability of normal intestinal cells. This technology could contribute to preventing tumor growth and metastasis while providing a patient compliance therapeutics, which will be crucial to the success of overcoming the challenges presented by cancers.

## 1. Introduction

Green chemistry and sustainable processes are nowadays paramount principles which industry must have present in their production lines [1]. The constant search for new methods with lower environmental risks is a transversal concern to the industry, and the pharmaceutical sector is no exception. For pharmaceutical and biomedical companies, particularly in the quest for new substances or carriers for drug delivery, to enhance the solubility or permeability, avoid polymorphism, decrease toxicity, and improve pharmacokinetics, green chemistry can provide powerful innovative solutions [2]. Nowadays, even though intense research and development are being undertaken, it is more and more difficult to synthesize new molecules with enhanced therapeutic efficacy to substitute the “old” ones. Consequently, pharmaceutical sciences and drug development research have been giving particular emphasis on the enhancement of existing drug properties.

## 2. Green Chemistry and Deep Eutectic Systems

In 1998, Warner and Anastas introduced the concept of green chemistry by listing its 12 principles. In organic synthesis and chemical engineering processes, the use of solvents should be minimized and solvents should be regarded only as auxiliary substances, hence being used in very low amounts. Whenever it is not possible to replace solvents completely and their use is required, they should be innocuous [3,4]. Consequently, in the past two decades, the research on safe and environmentally benign solvents has been increasingly growing. Several substances, such as water, ionic liquids (IL), supercritical fluids (SCF), and more recently deep eutectic systems (DES), have been considered as alternative green solvents [5,6,7,8,9]. DES were firstly reported in 2003 by Abbott and co-workers, as a novel class of green solvents with similar performance to IL [7,10,11,12]. Eutectic systems have been categorized in different classes according to the nature of their constituents. DES were first classified as type (I), a combination of a quaternarium salt with a non-hydrated metal halides; type (II), a combination of a quaternarium salt with a hydrated metal halides; type (III), a combination of a quaternarium salt (HBA) and a hydrogen bond donor (HBD); and type (IV), a combination of a metal chloride and a hydrogen bond donor [13]. Overall, all classes of DES encompass eutectic systems with a deep depression on the system’s melting point, when in comparison with its individual components, and a deviation from the ideality. After this first classification in these four types, other classifications and nomenclatures have been described. This is the case, for instance, of the term low transition temperature mixtures (LTTM), described by Francisco and co-workers, referring to mixtures of two or more compounds which include not only the systems with changes in their melting temperatures, but also associations of compounds which lead to any other changes on the thermal events in relation to the initial raw materials [14]. Under the definition of LTTM fall the well-known natural deep eutectic systems (NADES), which arose from the work developed in 2011 by Choi and co-workers. The preparation of more than 30 viscous liquids has been reported from combinations of sugars, natural carboxylic acids, choline chloride, and/or water [15,16]. In our daily life, we often handle NADES without even knowing it; honey and maple syrup are an example of that, in which mixtures of sugars form eutectic systems that are liquid at room temperature. Such examples may be used in food and nutritional supplements, but also in medical formulations, since they often present interesting bioactivities, are biodegradable, and present, most of the time, a low toxicity [13].

## 3. Therapeutic Deep Eutectic Systems

Therapeutic deep eutectic systems (THEDES) are described as eutectic systems in which one of the components is an API, or a system where active pharmaceutical ingredients (API) can be dispersed. A brief literature search in the ISI Web of Knowledge (03.08.2021) for “deep eutectic solvents” reveals 6040 references (590 reviews), with 59 references (18 of which reviews) on “deep eutectic systems pharmaceutics”, and in which Portugal is the leading country in the field.

The first studies reported in the literature refer to the possibility to dissolve API in DES. Stott and co-workers observed that DES could incorporate an API; therefore, DES acts as a delivery system while simultaneously improving the characteristics of the API itself [17,18]. Other molecules of interest to the pharmaceutical industry have, since then, been the object of study. Shen and co-workers focused on the use of a eutectic mixture of borneol:menthol for the intestinal absorption of daidzein [19]. Morrison et al. tested NADES as potential solubilization media for benzoic acid, griseofulvin, danazol, itraconazole, and AMG517, and the results showed a significant increase between 5 to 22,000-fold in the solubility of the API in NADES compared to water [20]. Li et al. have investigated other drugs, such as itraconazole, piroxicam, lidocaine, and posaconazole, and the solubility enhanced up to 6700-fold, 430-fold, 28-fold, and 6400-fold, respectively, when compared to their solubility in water [21]. Shekaari et al., in another study, analyzed the solubility enhancement of acetaminophen in NADES [22]. Goud et al. explored the formation of eutectic mixtures with curcumin, which could potentially be used as therapeutics for Alzheimer’s [23].

Several NADES and THEDES with different bioactivities have been investigated namely as antibacterial and antibiofilm, antifungal and anticancer agents, as well as wound healing enhancers. Table 1 summarizes some of the work carried out and the major aims of each study.

The use of THEDES revealed, hence, to be a powerful tool in pharmaceutical sciences enhancing the properties of existing drugs and contributing to the development of new formulations that could be more effective and easily administered [5]. However, this scientific area is still largely unexplored.

## 4. THEDES for Tuberculosis Treatment

**Figure 1:**
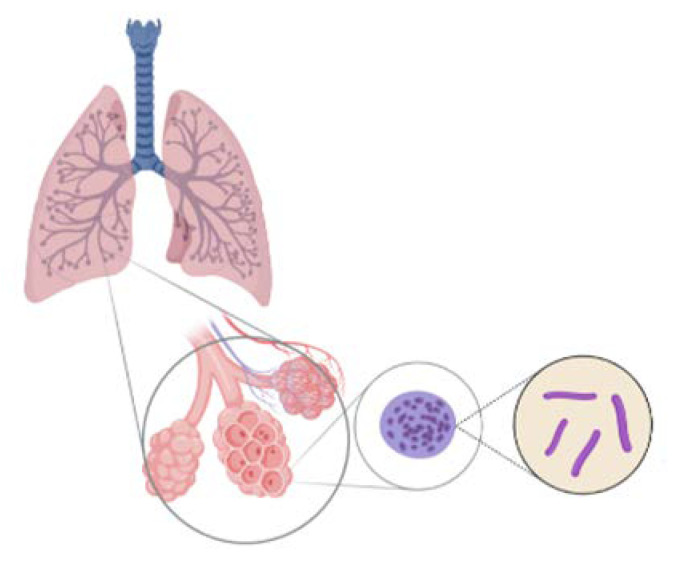
Schematic representation of the site of infection in TB. Created with BioRender.com.

Tuberculosis (TB) is described as an ancient disease that has been associated with humans at least since the Neolithic revolution and is characterized by its epidemic cycles with emergent outbreaks that then recede (Figure 1). Throughout modern history, TB has been associated with high density population areas living with poor sanitation. In Europe, TB had become an epidemic in the 18th and 19th centuries where it often revealed a seasonal incidence pattern. Public health measures, vaccination, and antibiotics (such as streptomycin) represented effective measures towards the control of TB. Nevertheless, the rise of drug resistant bacterial strains in the last three decades have push backwards the control and complete elimination of TB.

*Mycobacterium tuberculosis* (Mtb) is the cause of TB, and although it is considered a curable and preventive disease, it is still reported by the World Health Organization (WHO) as one of the deadliest infections worldwide, having killed an average of 1.4 million people in 2019 [37]. The WHO established policies to promote the decrease of the number of cases of TB and end with deaths triggered by this infection, encouraging the investigation of new drugs for TB treatment as well as more effective therapies. The WHO estimates that, for R&D, at least an extra US $1.1 billion per year is needed to accelerate the development of new tools for this problem [37]. Moreover, other factors, such as the recent pandemic caused by the coronavirus (SARS-CoV-2), can lead to a reversal of the progress made towards the decrease of global TB incidence, increasing its incidence and associated mortality [37]. Even though 14 vaccines, 22 drugs, and several combinatorial treatments are being studied in clinical trials, the drugs that present the best therapeutic outputs are still the same that were designed 50 years ago. Moreover, the indiscriminate use of antibiotics leads to the development of multi-resistant strains and consequent antibiotic resistance. The nonspecific antimicrobial activity of NADES is able to compromise the bacterial agents’ viability while avoiding the risk of inducing resistance.

In 2014, the WHO established a strategy to control and end TB by 2035 called the “End TB Strategy”, which is based in the development of sustainable goals, such as: (i) creation of integrated and patient-centered care and prevention strategies; (ii) creation of strong policies and supportive systems with participation of governments, communities and private investors; and (iii) promotion of the research and innovation to help accelerate the decline rates of global TB incidence and completely eliminate the TB epidemic. The anti-TB drugs that started to be investigated in the 1940s and in 1960s gave rise to a treatment that combines four of the first line anti-TB drugs and requires its administration for several months (a minimum of 6 months). This drug regimen is still the primary drug combination used as first-line treatment of TB and it is considered the more effective treatment available today.

THEDES have been used in research for pursuing improvements in TB therapy (Table 2). For instance, in our previous work we described an increase on the solubility of ethambutol and L-arginine when combined as a THEDES with citric acid [29]. Zakrewsky et al. reported a eutectic system based on choline bicarbonate and geranic acid (1:2 molar ratio) with antimicrobial activity towards Mtb drug-resistant strains [38]. Another study reported the use anti-TB drugs (pyrazinamide and isoniazid) to obtain binary and ternary solid eutectic compositions from a co-crystallization of these drugs with succinic acid and fumaric acid, showing that during co-crystallization synthesis the formation of eutectic mixtures could occur when cohesive interactions are predominant and the size/shape of the components are mismatched [39]. If the adhesive interactions dominating the resulting compound are characterized as a cocrystal, and yet if both interactions, adhesive and cohesive, are balanced and a match of size/shape between the components occurs, a solid solution is formed [39]. Furthermore, Rajbongshi et al. also studied the formation of eutectics through crystallization processes with pyrazinamide and different aromatic carboxylic acids characterized by a “V” shape phase diagram [40].

The study of eutectics for pharmaceutical applications have been increasing along the years and, particularly, in antimicrobial applications, such as for TB therapy, and has showed promising results with improvements in anti-TB drugs properties. However, little advances have been reported in the mechanisms underlying these observations. Monteiro and co-workers described a possible mechanism for the system Citric Acid:Ethambutol:H2O [41], unveiling a possible entry pathway for ethambutol promoted by citric acid through a recently published mycobacterial ABC (Rv1819c, PDB ID: 6TQE) transporter [42]. This transporter has been described to be involved in the uptake of hydrophilic compounds through the inner mycobacterial membrane. The results of this study suggest that the citric acid molecules interact with R287 while, at the same time, the negatively charged carboxylate groups strengthen the interaction between ethambutol and Rv1819c. Furthermore, the development of alternative formulations that can lead to new and more effective administration routes is another challenge in this quest for more efficient anti-TB therapies. The major problem in TB therapeutics is the long time needed for the treatment, which often leads to patients ceasing the treatment and, therefore, leading to the appearance of multi-resistant strains. One of the major concerns in TB treatment and prevention is related to drug-resistance that could emerge by spontaneous mutations of bacteria and trigger resistance to one or more effective anti-TB drugs, like the ones that are administered as first-line treatment such as isoniazid and rifampicin (multidrug-resistant tuberculosis, or rifampicin-resistant TB) [43,44] This problem could be even more serious when multidrug-resistant TB and drug-resistance to at least one of second-line anti-TB drugs occurs (extensively drug-resistant TB) [37,43,44] This may be even more severe when the bacteria do not respond to either the first-line or second-line of anti-TB drugs and are termed totally drug-resistant TB. These cases leave patients without additional treatment options that could be safe and effective [37]. Moreover, drugs used in second and third-line of treatment are, in general, less effective, more toxic, and comprise higher costs, as they will increase the time of the treatment for approximately 18–20 months. The immense variety of natural molecules accessible, coupled with the advantages of designing tailor-made systems, has led to the development of several NADES applications in the past years, particularly working as promising antimicrobial agents and promoters of biofilm detachment. Several groups have reported the antimicrobial activity of eutectic systems, however, most of them do not rely solely on natural occurring molecules. Silva and co-workers revealed the use of fatty acids, such as capric acid, myristic acid, stearic acid, and lauric acid in NADES formulation, rendering systems with antimicrobial properties. The most promising system towards Gram-positive bacteria and C. albicans was capric acid:lauric acid (2:1). This system seems to be highly interesting as well for the detachment of biofilms of several types of microorganisms [32]. Giving the high incidence and mortality of TB, alongside with all the research that has been carried out to discover effective treatments, exploring combinatorial approaches might be the path to follow. It became evident that combinatorial therapies are able to achieve target-specificity, increased bioavailability, provide lower toxicity towards normal tissues, promote drug controlled released, and are efficient in formulation for a specific route of administration while in compliance with the green chemistry metrics. One example of combinatorial technologies associates the use of THEDES design for TB therapy and SCF technology. This work, presented by our group, describes the preparation of THEDES with L-Arginine (described as an adjuvant in TB therapy) and the design of inhalable particles through SCF technology (PGSS), achieving an encapsulation efficiency of approximately 75%, revealing promising results for encapsulation of THEDES [30]. This is just one example of what could be achieved by combining different strategies like nanotechnology with green solvents and/or SCF technology for the formulation of nanoparticles in TB therapy.

## 5. THEDES for Colorectal Cancer Treatment

**Figure 2:**
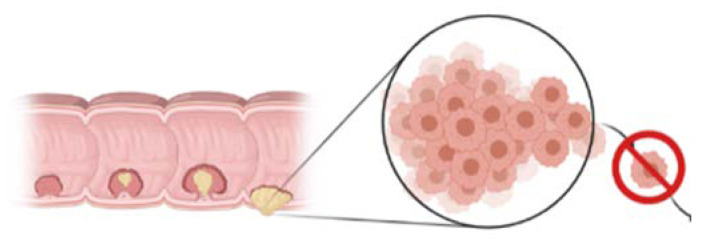
Schematic representation of progression of colorectal cancer (CRC). Created with BioRender.com.

Cancer is also a high burden economic and social devastating disease, in which mortality has been comparatively rising and has already overcome cardiovascular diseases as the main cause of dead in certain countries [45]. According to the WHO, in 2019 cancer was responsible for almost 10 million deaths worldwide, indicating that one in every six deaths is cancer related [46]. This sharp increase is due to several key factors, such as infections, chronic malnutrition, lack of access to medical care, but also population aging and unhealthy lifestyle choices, making cancer a burden both for the rapidly developing countries but also developed ones. Colorectal cancer (CRC) is the third most commonly diagnosed cancer and, if detected early and treated accordingly, has a high cure rate [46]. Nonetheless, it occupies the second position in the deadliest cancers [46]. Therefore, the high mortality rate and poor prognosis of CRC reveals the ineffectiveness of current therapies in controlling the disease progression, particularly in the case of advanced or metastatic phases (Figure 2). Thus, the need for alternative approaches to eliminate premalignant lesions, control their progression to cancer, and to avoid cancer recurrence are of at most importance.

The cytotoxicity of different NADES based on ammonium- and choline chloride for cell lines, such as human colon adenocarcinoma cell line (HT29), human malignant melanoma cell line (A375), among others, has been reported [47]. Table 3 presents a summary of the THEDES systems together with their reported anti-cancer activity.

**Figure 3:**
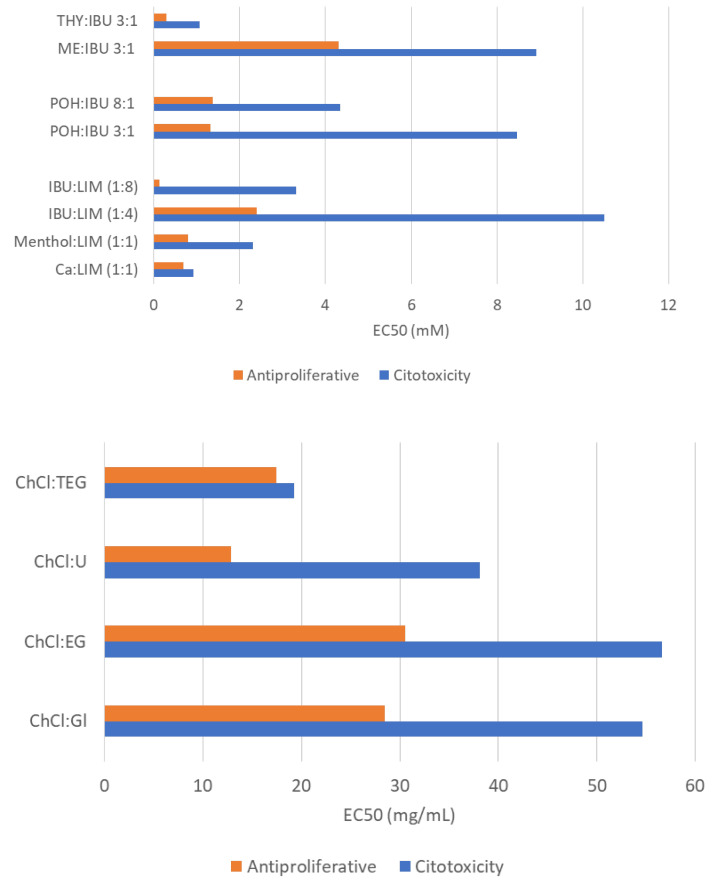
THEDES cytotoxicity and antiproliferative activity measured as the effective concentration to inhibit 50% of cell growth (EC_50_).

The anticancer activity of THEDES has to be evaluated against the effect of the systems in so-called “normal” cell lines, i.e., cell lines which mimic a healthy environment. Figure 3 represents the EC_50_ values reported for the different systems, in which their cytotoxicity is concerned, i.e., effective concentration to reduce cell viability in 50%, towards healthy cells and their antiproliferative activity towards cancer cells.

The mechanisms of action of the systems with anti-cancer reported activity are still unclear, despite some hints that have been disclosed. For example, Mbous and co-workers report that NADESs activity, namely choline chloride:fructose, choline chloride:glucose and *N*,*N*-diethyl ethanolammonium chloride:triethylene glycol, by increasing membrane porosity and redox stress [47].

When it comes to targeting CRC cells, terpenes such as limonene, perillyl alcohol, menthol, and thymol, amongst others, combined with non-steroidal anti-inflammatory drugs (NSAID) have been studied and have shown great promise. Interestingly, it has been demonstrated that, when compared to its individual components, a eutectic mixture results in a new entity with different and enhanced properties, thus causing a more selective and severe impact on cancer cells [47]. However, the specific cytotoxic action of THEDES towards both malignant and non-malignant cells is still unclear [34,47,50]. These combinations with NSAIDs arise from the finding that NSAIDs reduce CRC and tumor recurrence risk by 40–50% [51]. Terpenes are plant secondary metabolites which may have an important role in the pursuit of natural molecules with anticancer potential by relying on their displayed cytotoxic action. Among the cancer hallmarks, there is the sustaining of proliferative signals and resisting cell death. In CRC, limonene has been reported to induce apoptosis via mitochondrial pathways and affecting the PI3K/Akt signaling pathway for survival and apoptosis [52]. Our first studies, as reported by Pereira and co-workers, have demonstrated that IBU:LIM (1:4) has a dose-dependent effect and that the mechanism of action is completely different from isolated IBU and LIM. Furthermore, the assessment of intracellular reactive oxygen species (ROS) and nitric oxide (NO) production indicate that the lowest tested concentration of IBU:LIM (1:4) was able to protect HT29 cells from oxidative stress, showing anti-inflammatory effects through the inhibition of ROS and NO production [34]. Perillyl alcohol, menthol, and thymol are other examples of terpenes with an associative cytotoxic action towards cancer cells. In CRC, the epithelial–mesenchymal transition is widely associated with angiogenesis and a chronic inflammatory state [53]. Cox-2 is upregulated in CRC and the production of Cox-2-derived prostaglandin is known to promote β-catenin activation [53]. Moreover, it has been reported in the literature that the aberrant activation of the (Wnt)/β-catenin signaling pathway is known to be responsible for approximately 90% of CRC cases [54,55]. β-catenin plays an important role in cell-to-cell adhesion and gene transcription regulation [55]. Only through the understanding of the specific cytotoxic action of THEDES towards both malignant and non-malignant cells will be possible to design adequate delivery systems to successfully target CRC and provide a significant contribution to decrease the associated number of deaths.

## 6. Conclusions and Outlook

In pharmaceutical sciences, therapeutic deep eutectic systems are still in their infancy and exciting developments may be foreseen in the coming years. The studies carried out so far pave the way towards new findings, particularly those concerning the enhancement of drug bioavailability, either by increasing the solubility of the API in aqueous solutions or by increasing its permeability through relevant biological barriers (skin, intestinal wall, or other biological tissues). Side by side, with these advances go along new findings on the molecular understanding of the interactions that take place. From the formation of hydrotopes to enhance the API solubility, to the interactions of the THEDES with biological membranes and tissues, major advances are to be expected when these interactions are better understood. With regards to the advances in particular therapeutic applications, such as TB and CRC, which are the ones described in this manuscript, together with the advances in the fundamental knowledge on deep eutectic systems, the understating of the mechanisms of action of these liquid formulations and their differences in comparison to the solid APIs will help boost the adoption of these green technology platform by the pharmaceutical industry.

## Figures and Tables

**Table 1:** Summary of different eutectic systems reported in the literature and their aim.

THEDES	Aim	Reference
Choline Chloride:Acetylsalicylic Acid Choline Chloride:Phenylacetic Acid Menthol:Benzoic Acid Menthol:Acetylsalicylic Acid Menthol:Phenylacetic Acid	Preparation of THEDES to study their bioavailability	[24,25]
Menthol:Ibuprofen	Incorporation of THEDES with NSAID in a polymeric matrix	[26]
Choline Chloride:Mandelic Acid	THEDES preparation and encapsulation in fiber membranes	[27]
Choline Chloride:Ascorbic Acid	Preparation of NADES and enhancement of dexamethasone solubility	[28]
Citric Acid:Ethambutol:H_2_O	Preparation of THEDES with API for TB	[29]
Citric Acid:L-Arginine:H_2_O	Encapsulation of THEDES in a lipidic carrier	[30]
Menthol:Lauric Acid Menthol:Myristic Acid Menthol:Stearic Acid	Development of THEDES with terpenes and fatty acids with antimicrobial and wound healing properties	[31]
Lauric Acid:Myristic Acid	Preparation of eutectic blends and loading onto gauzes	[32]
Capric Acid:Lauric acid Capric Acid:Stearic Acid Capric Acid:Myristic Acid	Preparation of different DES with fatty acids with antimicrobial activity and biofilm detachment	[33]
Limonene:ibuprofen Perillyl alcohol:ibuprofen Menthol: ibuprofen Thymol:ibuprofen	Preparation of THEDES with terpenes + NSAIDS and evaluation of their activity against CRC cells	[34,35,36]

**Table 2:** Summary of the THEDES prepared for anti-tuberculosis treatment.

THEDES	Reference
Citric Acid:Ethambutol:H_2_O	[25]
Choline bicarbonate: geranic acid (1:2)	[38]
Pyrazinamide:succinic acid Isoniazid:succinic acid	[39]
Pyrazinamide: Nicotinic acid (2:1) Pyrazinamide:isonicotinic acid (5:1) Pyrazinamide: saccharin (4:1)	[37,40]

**Table 3:** Summary of reported THEDES with anticancer activity.

THEDES	Reported anti-cancer activity	Reference
Choline Chloride:Glycerine Choline Chloride: Ethylene glycol Choline Chloride:Urea Choline Chloride:Triethylene glycol	Human breast cancer cell line (MCF-7); Human prostate cancer cell line (PC3); Human malignant melanoma cell line (A375); Human liver hepatocellular cell line (HepG2); Human colon adenocarcinoma cell line (HT29); Carcinoma-derived human oral keratinocyte cells (H413)	[48]
Choline Chloride:fructose Choline Chloride:glucose *N*,*N*-diethylethanol ammonium chloride:Triethylene glycol	MCF-7, PC3, A375, Human gastric adenocarcinoma (AGS), Human uterus adenocarcinoma (HelaS3), Human hepatic cell lines (WRL-68)	[47]
Piperidinyl:diethylstilbestrol Pyrrolidinyl:diethylstilbestrol	Human Breast Carcinoma cells (MCF-7)	[49]
Limonene:ibuprofen Perillyl alcohol:ibuprofen Menthol: ibuprofen Thymol:ibuprofen	HT29	[30,31,32]

## Data Availability

Not applicable.

## Sample Availability

Samples of the oregano oil used in this study are available from the authors.

## Abbreviations

API	Active pharmaceutical ingredient
CRC	Colorectal cancer
DES	Deep eutectic system
HBA	Hydrogen bond acceptor
HBD	Hydrogen bond donor
IL	Ionic liquid
LTTM	Low transition temperature mixtures
Mtb	Mycobacterium tuberculosis
NADES	Natural deep eutectic system
NO	Nitric oxide
NSAID	Non-steroidal anti-inflammatory drug
PGSS	Particles from gas saturated solutions
ROS	Reactive oxygen species
TB	Tuberculosis
THEDES	Therapeutic deep eutectic system
SCF	Supercritical fluids
WHO	World Health Organization
